# High-Performance Breaking and Intelligent of Miniature Circuit Breakers

**DOI:** 10.3390/s22165990

**Published:** 2022-08-11

**Authors:** Jianning Yin, Xiaojian Lang, Haotian Xu, Jiandong Duan

**Affiliations:** 1 School of Electrical Engineering, Xi’an University of Technology, Xi’an 710048, China; 2 Zhejiang Tengen Electric Co., Ltd., Yueqing 325604, China

**Keywords:** DC interrupting, digitization, remote control, electric energy measurement, miniature circuit breaker

## Abstract

The exploitation and utilization of clean energy such as wind and photovoltaic power plays an important role in the reduction in carbon emissions to achieve the goal of “emission peak and carbon neutral”, but such a quantity of clean energy accessing the electric system will foster the transition of the electric power system structure. The intelligentization of power equipment will be an inevitable trend of development. High breaking performance, remote control and a digital detection platform of miniature circuit breaker, a protective equipment of a power distribution system, have also been inevitable requirements of the power Iot system. Based on the above, this paper studies three aspects: high-performance AC and DC general switching technology, remote control technology and operation status’ digital monitoring. A new DC non-polar breaking technology is proposed, which improves the short circuit breaking ability. An experimental prototype using the above techniques was fabricated and passed the DC 1000 V/10 kA short-circuit breaking test. On the basis of the above, an intelligent circuit breaker is developed, which contains multiple functions: remote switching, real-time temperature detection, energy metering and fault warning. Moreover, a software for digital condition monitoring and remote control is developed. This work has certain theoretical and practical significance for the development of the power Internet of things.

## 1. Introduction

Today, the regulation of the global pollution has been urgent, especially massive quantities of carbon dioxide emissions which lead to a more serious greenhouse effect and higher sea level that are threatening the global environment. Therefore, it has been a Global Common Mission to reduce the carbon emissions. China has also put forward the long-term vision of “emission peak and carbon neutral”, and the raising of the goal will necessitate the exploitation and utilization of clean energy. Such substantial quantities of distributed clean energy accessing the electric system will foster the transition of the power system framework and equipment. Aiming to adequately absorb clean energy, it has been a trend of electric system reform to construct a new power system taking clean energy as the main body, and the promised power system will be more intelligent, shared and controllable [1,2]. At the same time, smart grid and distributed power technology will also boom. The digitalization and intellectualization of the core power equipment among the new power system will hoist visualization, regulation capacity and promote consumption utilization level of new energy generation, so as to speed up the transformation from power grid to energy internet; electrical equipment’s intellectualization and digitalization endow its status with visualization, so as to support a more flexible and moderate energy rationing platform [3,4]. Digitalization of power distribution allows facility managers and maintainers to efficiently solve problems with less energy, reducing operating and maintenance expenses [5].

Acting as the core equipment of the power distribution system’s terminal protection and regulation, MCB (miniature circuit breaker)’s intellectual trend promotes the achievement of power distribution digitalization [6]. At the same time, the exploitation and utilization of new energy generation, especially the boom of photovoltaic power, raises higher requirements to the DC current breaking capacity of MCB. With the development of AC–DC hybrid distribution network, high voltage (1000 V) and AC/DC universal MCB faces a great challenges.

The research on the breaking ability and the invention of new products concerning MCB have been going on for many years. A large number of scholars have conducted thorough research on its arc extinguishing ability [7,8]. Most of the research focuses on arc characteristics. There are two main schemes to study the arc characteristics of the circuit breaker: simulation and experiment [9,10,11,12,13,14]. For simulation, magneto-hydrodynamics (MHD) has become an effective auxiliary mean to study characteristics of arc’s motion and vanishing [15,16,17,18]; for experiment, the arc’s diagnostic means mainly include optical fiber testing, high-speed photography and laser light filling. The dynamic characteristics and breaking performance of arc via recording the arc motion form are studied [19,20,21]. Most of the DC MCB products on the market are below 1000 V, and the breaking capacity is insufficient (6 kA) and unstable. Regarding remote control and digitalization, as the technology of the Internet of things experiences continuous development in recent years, remote monitoring technology based on WIFI/4G is proposed [22]. Some industry conducted preliminary studies on the intelligent circuit breaker and developed some of those products. However, there are few circuit breakers that can realize electric energy measurement and real-time online monitoring of operating status [23,24]. Therefore, it is of great theoretical and practical significance to study the remote control, digitalization and AC/DC general high-performance breaking technology. Based on this, the DC non-polar breaking technology of MCB is studied firstly, and an arc extinguishing strategy of coordinated control of magnetic blowing and air blowing is proposed. An experimental prototype using the above techniques was fabricated and passed the DC 1000 V/10 kA short-circuit breaking test. Secondly, the intelligent technology of MCB is studied. The integrating electric operation mechanism to realize remote control, visualization system, digital monitoring platform and mobile application (APP) based on a cloud platform are developed. The remote control, temperature monitoring, power management, automatic alarm and real-time monitoring of MCB are realized. The safe and efficient operation of load and terminal network is enhanced. The above research contributes to the development of a new power system via providing theoretical and technical reference for the improvement and optimization of system protection appliances after the high proportion of new energy access.

This paper is organized as follows: Section 2 presents high-performance (1000 V/10 kA) DC/AC breaking technology, and the principle and experimental results are introduced. Section 3 presents intelligent technologies of MCB, and the prototype realization principle and product performance are introduced. Section 4 is the conclusion.

## 2. High-Performance DC/AC Breaking Technology

For the breaking characteristics of a micro-circuit breaker, whether the circuit breaker can be successfully broken is directly determined by whether the air arc can be extinguished smoothly and quickly. Because of the inexistence of natural zero point, a DC arc is more difficult to put out compared with that of AC, and its breaking ability cannot be improved via increasing the distances and quantities of open gate pieces or any other conventional technologies due to the limitation of small volume of MCB; furthermore, single arc extinguishing measures have been unable to meet the higher voltage (1000 V) of the DC circuit breaker open performance requirements. At the same time, the requirements of power system distribution equipment hope the DC circuit breaker can realize the non-polarity breaking, so the structure design of its arc extinguishing chamber is facing tougher challenges.

In order to enhance the energy dissipation of the arc, this paper puts forward an arc extinguishing strategy coordinated by air blowing and magnetic blowing, so as to realize the purpose of breaking large DC current with a small volume by increasing the effect of air blowing and magnetic blowing in the arc extinguishing chamber.

### 2.1. Theoretical Analysis and Practical Scheme of DC Non-Polar Arc Extinguishing

**Figure 1:**
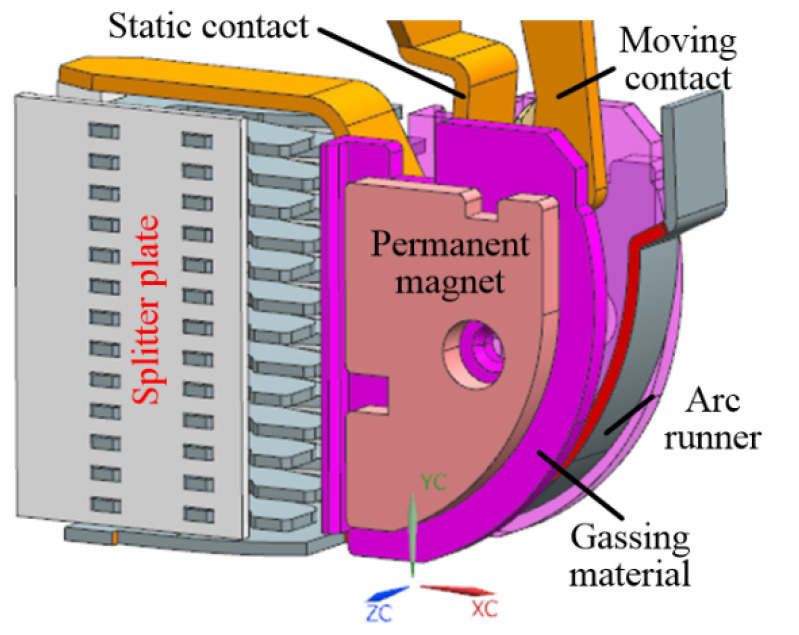
Layout scheme of arc extinguishing chamber.

**Figure 2:**
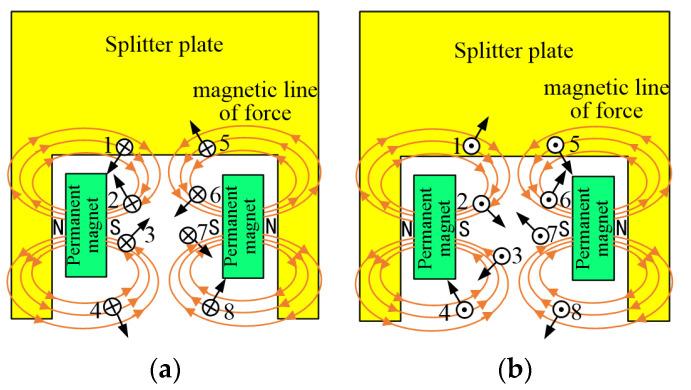
Arrangement schematic diagram of permanent magnet. (**a**) The current goes into the paper. (**b**) The current goes out of the paper.

The traditional arc extinguishing strategy has been difficult to match the high voltage DC interruption. Therefore, an arc extinguishing scheme with coordinated control of magnetic blowing and air blowing is proposed. Specifically, the permanent magnets and gas-producing materials are added to the arc extinguishing chamber: on the one hand, permanent magnet is used to enhance the magnetic blowing effect; on the other hand, gas generation material is used to enhance the air blowing effect. The overall layout scheme of the arc extinguishing chamber is shown in Figure 1. Permanent magnets are placed on both sides of the contact and arc running area, respectively, and the gas-producing material is wrapped on the outside of the permanent magnet. On the one hand, the air blowing can be enhanced, and on the other hand, the permanent magnet can be prevented from losing magnetism due to direct contact with the high-temperature arc. Due to the polarity of permanent magnets, it is a necessity to arrange the position of permanent magnets reasonably in order to realize the non-polar breaking of DC arc. The schematic diagram of non-polar permanent magnet layout scheme is shown in Figure 2. Figure 2 shows the schematic diagram of the arrangement of two permanent magnets’ S poles opposite each other. In order to realize the DC non-polar breaking, the permanent magnets are arranged on both sides of the arc extinguishing chamber wall, respectively, with the same magnetic poles being opposite with each other.

The extinguishing of the DC arc mainly depends on the current limiting of the arc voltage, which forces the current to cross zero to extinguish. Therefore, increasing the arc voltage is the fundamental measure for arc extinguishing. Increasing the arc voltage mainly depends on the splitter plate cutting the arc, forming multiple near-pole voltage drops. Therefore, the main measures in the arc extinguishing design are to make the arc enter the splitter plate area quickly. For DC non-polar breaking, that is, after the current direction is changed, it does not affect the smooth entry of the arc into the arc extinguishing chamber.

Figure 2a shows the direction of the Loren magnetic force in the arc columns at eight different locations when the current direction is straight into the page. It can be seen from the figure that when arc column is located in the point 2 or 5, the Loren magnetic force moves arc to left and up direction; when arc column is located in the point 3 or 8, the Loren magnetic force moves arc to right and up direction, which is of benefit for the arc being blown into the grid, cooled and cut so as to improve the arc voltage.

When the arc column is located at point 1, it will move to the point 2 under the action of Loren magnetic force, and the changed Loren magnetic force will move arc to right and up direction and push it into grid region; when the arc column is located at point 6, it will move to the point 3 and receive the force to the upper right of the grid; when the arc column is located at point 7, it will move to the point 8, and the Loren magnetic force will move arc to right and up direction and push it into grid region. When the arc column is located at point 4, on the one hand, Loren magnetic force will move the arc toward the contact area; on the other hand, the presence of gas producing materials will force arc toward the grid area, and, due to the complex interaction, the arc will eventually move toward the grid area.

From the above analysis, it can be seen that the arc can enter the grid area and be cut quickly no matter how the direction of current place. At the same time, the permanent magnet arrangement scheme shown in Figure 2 can reduce the pinch force of the magnetic field generated by the arc itself and weaken the hindering effect of the magnetic field on the arc column movement, so as to further hasten the speed of that entering the grid area, promote the rapid rise of the arc voltage and improve the DC arc breaking ability.

### 2.2. DC Breaking Test and Result Analysis

(1) Test prototype and conditions.

**Figure 3:**
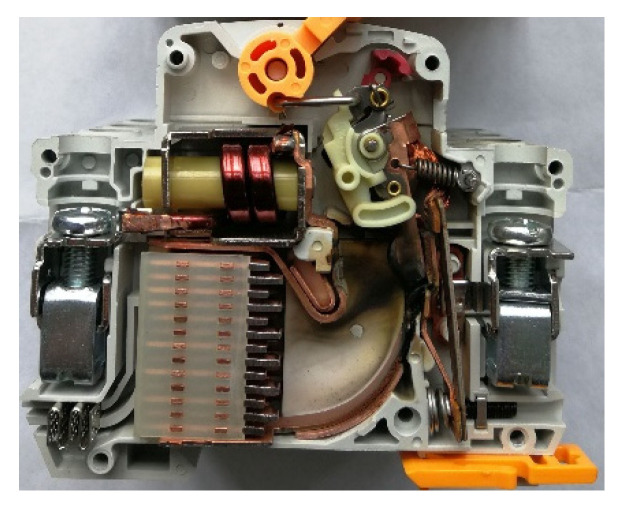
Experimental prototype.

Based on the above theory, a MCB test prototype is made, as shown in Figure 3: The permanent magnet is arranged on both sides of the contact and arc running area and wrapped by the gas-producing material. In order to verify the breaking ability of this scheme, the test was carried out under the conditions of 1000 V DC, 10 kA short-circuit current and 5 ms time constant. The test was carried out in the standard circuit breaker test station.

(2) Analysis of test results.

**Figure 4:**
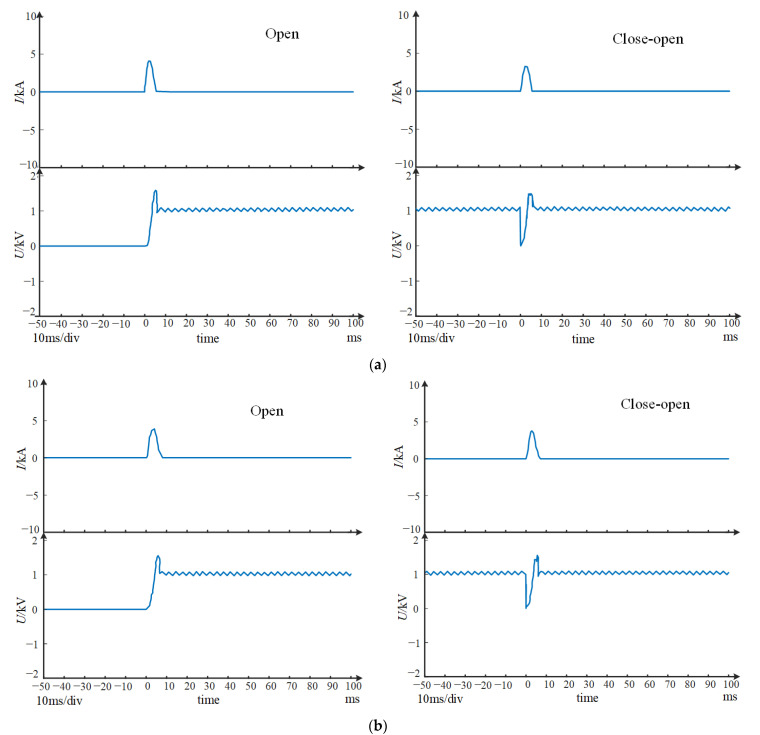
DC breaking test waveform (third party test). (**a**) The waveform of forward connection. (**b**) The waveform of reverse connection.

In order to verify the non-polarity breaking capacity of this scheme, a prototype of forward connection and reverse connection was tested in the short-circuit experiment. According to the short-circuit breaking capacity test standard of circuit breakers, an o (open)-co (close-open) standard process needs to be completed under short-circuit current. That is to say, one experiment is closed before power-on and opened directly after power-on, and the second time is closed and then opened after power-on. DC breaking waveform (including the arc current and voltage curves) is shown in Figure 4.

It can be seen from the arc voltage waveform that the arc voltage rises rapidly, and the highest voltage is more than 1800 V, which exceeds the system voltage 1000 V to a large extent. At the same time, the arcing time is about 5 ms. This is mainly because the arc quickly enters the splitter plate area and is cut by the splitter plate under the combined action of magnetic blowing and air blowing. At the same time the dissipation of arc energy is enhanced. Under the joint action, the arc voltage rises rapidly, and the current limiting effect is obvious. Therefore, the arc current quickly crosses zero and extinguishes, shortening the arcing time.

As it can be seen from the above test waveform, whether it is a forward or reverse connection, the circuit breaker prototype has successfully broken the short circuit current of 10 kA, and shortened the arc burning time, which fully verifies that the above permanent magnet layout scheme can achieve non-polar breaking and improve the breaking capacity compared with that of the market conventional circuit breaker (6 kA). The results show that the magnetic blowing and air blowing coordinated control strategy is effective in improving the breaking capacity.

The coordinated control strategy of arcing above does not need to change the size of the original circuit breaker and the main structure but only needs to place the permanent magnets and gas material on both sides of contact and arc running area; therefore, the scheme can not only be used for the development of high-performance DC circuit breaker but can also be applied to existing AC–DC optimal design of the miniature circuit breaker directly, in order to enhance the breaking capacity of short-circuit current.

## 3. Intellectualization of MCB

With the development of Internet of things technology, intelligent and digital requirements are put forward for the MCB used in the distribution system terminal. In order to realize the remote opening and closing control and online status monitoring of the MCB, the hardware and software systems are researched and developed.

### 3.1. Intelligent Platform Architecture

**Figure 5:**
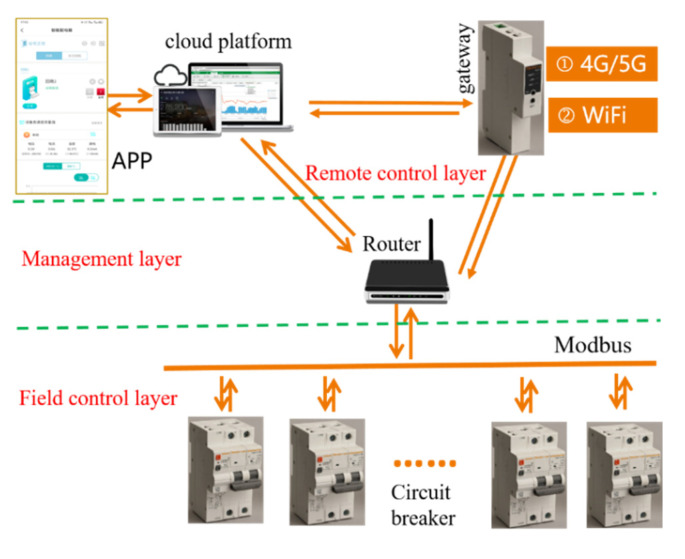
Intelligent overall architecture of circuit breaker.

The intelligent platform architecture of MCB is shown in Figure 5. The APP is an abbreviation of “mobile phone application”. The remote control is mainly based on the cloud platform, and the communication between the cloud platform and the circuit breaker is realized through the gateway. The gateway is connected with the circuit breaker module by a Type C data cable and can be configured with WIFI and data networks. At the same time, the development of a web version and a portable mobile phone APP realize the digital monitoring of the circuit breaker running state, remote opening and closing through the operation of the APP, power managing, temperature monitoring, overtemperature alarm, automatic trip and other multiple functions.

In order to achieve remote control, it is necessary to configure the circuit breaker hardware as follows: through the operating mechanism to realize the opening and closing operation, through the voltage and current sensor to realize the data acquisition and power management and through the temperature sensor to realize the real-time monitoring of the temperature of the circuit breaker.

### 3.2. Intelligent and Digital Circuit Breaker System

**Figure 6:**
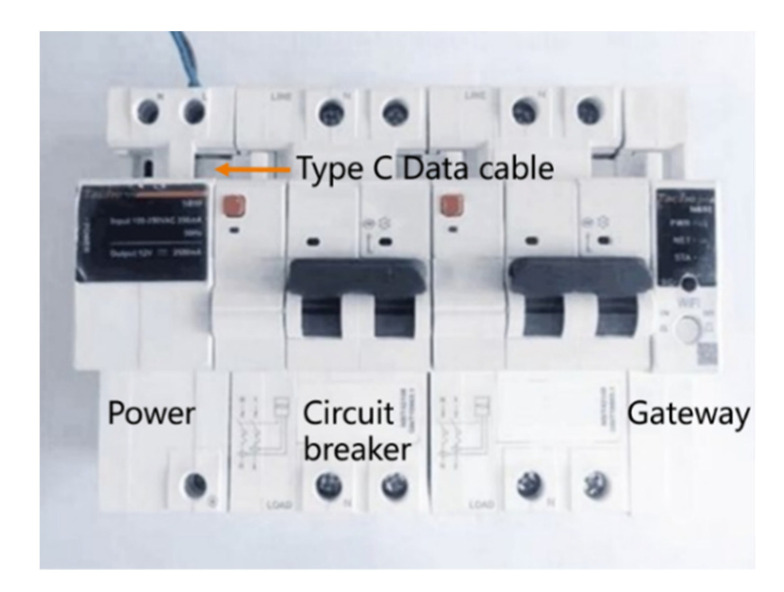
Composition of intelligent MCB.

The whole intelligent circuit breaker is shown in Figure 6, which mainly consists of three modules: power module, gateway and circuit breaker module. The three modules are connected by a Type C data cable. The input of the power module is AC 220 V, and the output is DC 12 V, whose main function is to supply power to the gateway and single chip operating mechanism of the circuit breaker module. The gateway plays the role of network communication; the Type C cable not only provides power but also implements communication for the gateway and the circuit breaker modules.

**Figure 7:**
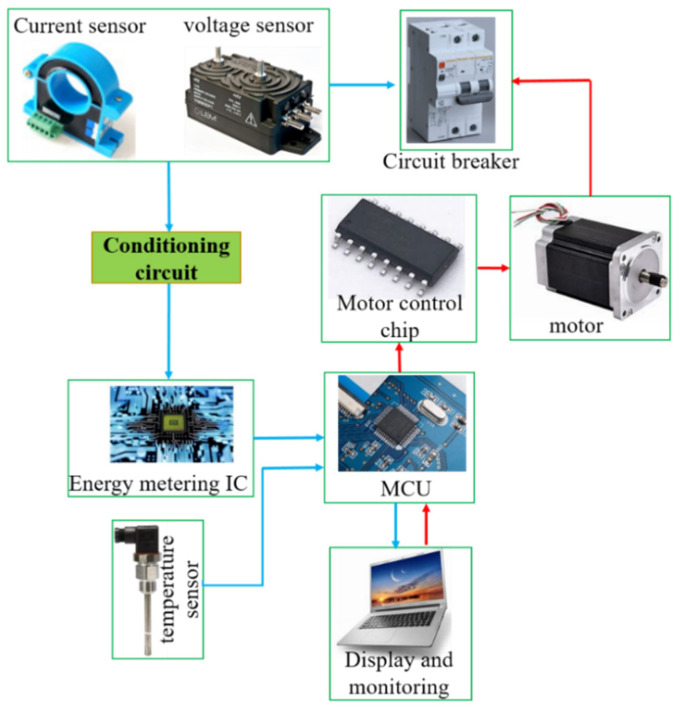
Diagram of hardware system.

Compared with the conventional circuit breaker, the new intelligent micro-circuit breaker products share the circuit breaker module plus a pole, used to install operating mechanism, control board, sensors and other devices to build a digital monitoring platform. The hardware system mainly includes a data acquisition system, central processor, actuator, display unit and circuit breaker. The hardware system diagram is shown in Figure 7.

(1) Remote control opening and closing technology.

The “conditioning circuit” mainly filters the arc voltage and current signals. The MCU is the Single Chip Microcomputer MKE02Z. The main function is to process and display the collected data and issue opening and closing instructions to the motor control chip.

**Figure 8:**
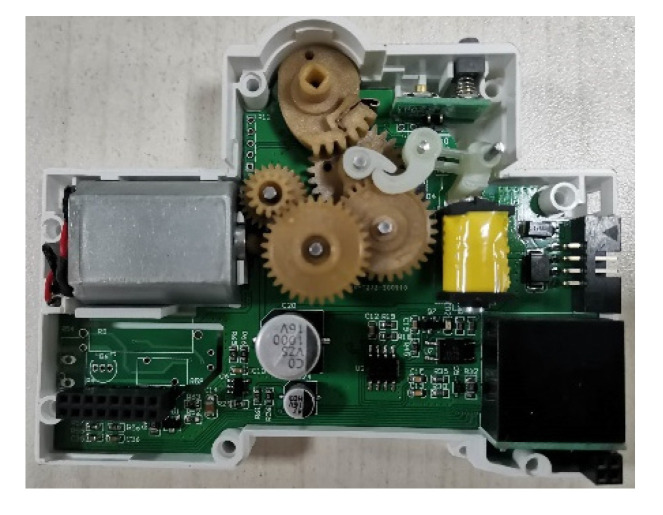
Hardware of operating mechanism and data processing and acquisition.

In order to realize the software remote control circuit breaker opening and closing, the hardware system added a motor with a control chip. When switching remote control points, firstly choose circuit breaker which requires operation in the display interface or the phone APP interface, clicking on the corresponding button; then issue instructions to the chip which control the motor turning forward or reverse by the single chip micro-computer; then drive the gears which link the circuit breaker handle with coaxial connection, so as to realize the remote points and closing operation. The entire operating mechanism and data acquisition hardware layout are shown in Figure 8.

(2) Data acquisition and digital display interface.

In order to realize the digital monitoring of running status of the circuit breaker, the hardware system uses an NXP single-chip micro-computer as the main control chip, installed the voltage sensor, current transformer and temperature sensor to complete the data collection of voltage, current and temperature. In order to realize the electric energy metering function, the voltage and current signals are sent into the electric energy metering chip through the conditioning circuit to complete the consumption calculation, then are sent into the digital display interface through the MCU main control chip to realize the digital real-time display of voltage, current and electric energy.

Moreover, the single-chip computer stores and processes data. When the circuit breaker’s real-time temperature exceeds its rated temperature, the main control chip will emit alarm instructions, on the one hand displaying alarm information on popup windows in the system, on the other hand conveying fault signal to the circuit breaker failure indicator and control its flashing, so as to realize real-time fault alarm, facilitating maintenance personnel.

**Figure 9:**
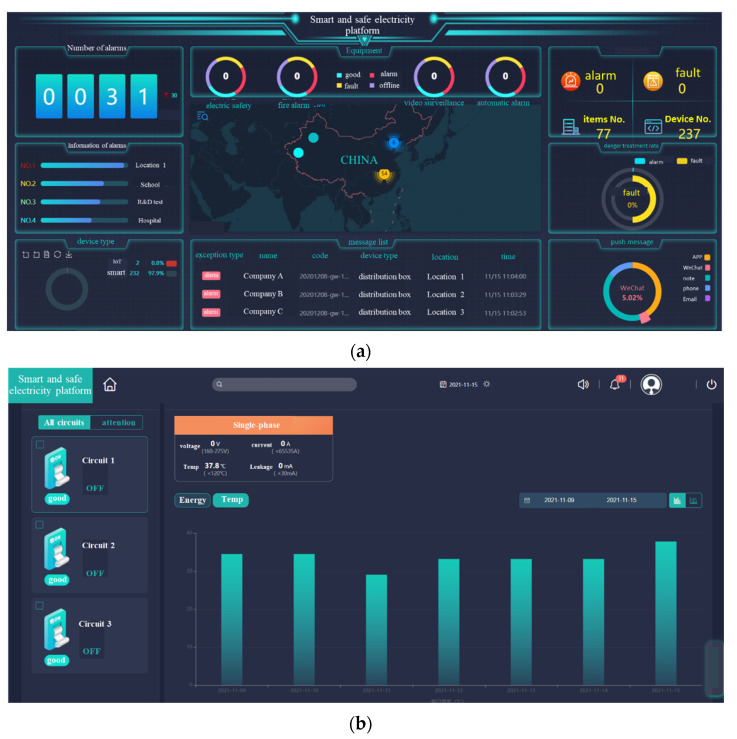
Circuit breaker digital monitoring system. (**a**) The main interface. (**b**) Circuit breaker monitoring interface.

Based on the above principles, the corresponding monitoring system and APP were developed. The interface of monitoring system is shown in Figure 9. The number of circuit breakers and specific information in different states (include good, alarm, fault and offline) are displayed in the interface. At the same time, the fault recognition rate, push information method, etc. are also displayed on the interface. Opening and closing commands can be issued through the circuit breaker monitoring interface and display the operating status of the circuit breaker. The temperature and power of each circuit breaker can also be viewed in real time through the interface.

Through this monitoring system, all circuit breaker layout points and operation data in the whole process can be digitally monitored. At the same time, the system can be used for a specific circuit breaker to achieve remote open and close operation, electric energy measurement, current and voltage monitoring, circuit breaker operating temperature display, real-time warning of overtemperature and so on. In the opening and closing operation, one circuit breaker can be operated alone, and multiple channels can be operated at the same time. Voice control is added in the mobile phone APP.

The digital control system above endows the power distribution system terminal protection equipment MCB with intelligent and digital monitoring and endows it with the functions of remote opening and closing, electric energy measurement, over-temperature alarm and so on, which facilitates the operation of users and improves the reliability of power equipment. At the same time, it can be seen that the current digital monitoring of the circuit breaker is still on a computer or mobile phone APP, with limited functions, requiring further research and development aiming digital control pane, so as to realize the integrated design of circuit breaker and digital monitoring platform, providing theoretical foundation for research of new generations of digital circuit breakers.

In combination with the high-performance arc open-circuit technology and intelligent technology mentioned above, the prototype was made and successfully passed through the third party test, providing technical reference for intellectualization and digitalization of power equipment in China, providing reference for the research and development of a high-performance DC intelligent circuit breaker for photovoltaic power generation, intelligent park and energy storage system.

## 4. Conclusions

(1) The arc extinguishing strategy coordinated by air blowing and magnetic blowing was proposed. The high-voltage DC non-polar breaking capacity of circuit breaker was improved, which increased from 6 kA to 10 kA DC.
(2) Through the reasonable arrangement of permanent magnet, this arc extinguishing scheme can realize DC non-polar breaking, AC and DC universal, while keeping the original circuit breaker structure size unchanged. The arc-extinguishing scheme can be extended to the research and development of high-performance DC molded case circuit breakers and frame circuit breakers.
(3) Based on the cloud platform, the intelligent and digital monitoring system of circuit breaker was developed, which provides a reference for the digitalization of power equipment.
(4) Through the combination of the above high-performance breaking technology and intelligent technology, the prototype was developed, confirming this technology has formed a mature product and has been promoted and transformed.
